# Perceived Neighborhood Social Cohesion is Associated with Lower Depressive Symptoms among Older Adults Diagnosed with Obstructive Sleep Apnea

**DOI:** 10.1002/alz70858_107620

**Published:** 2025-12-26

**Authors:** Erin‐Leigh Gallop

**Affiliations:** ^1^ University of Miami, Miami, FL, USA

## Abstract

**Background:**

The present study examines the intriguing relationship between perceived neighborhood environments and depressive symptoms in older adults diagnosed with obstructive sleep apnea (OSA), defined by an apnea‐hypopnea of five or more episodes. Identifying the risk factors for depressive symptoms is paramount as it has the potential to improve the quality of life for individuals in this vulnerable group. This study examines the relationship between perceived neighborhood social cohesion and depressive symptoms among older adults with an obstructive sleep apnea (OSA) diagnosis.

**Research Design and Methods:**

Data were derived from a subsample of older adults with a doctor's diagnosis of OSA from the Health and Retirement Study (*N* = 472), a nationally representative, population‐based study of older adults conducted in 2016. Our outcome, depressive symptoms, derives from eight items from the Center for Epidemiological Studies‐Depression scale. Our independent variable, perceived neighborhood social cohesion, is based on a four‐item, previously validated scale. Negative binomial regression models were used to examine the relationship between neighborhood social cohesion and depressive symptoms among older adults with an OSA diagnosis, adjusting for demographic factors, neighborhood disorder, smoking, drinking, chronic conditions, health insurance, medication usage, and insurance policies.

**Results:**

After adjusting for demographic factors, higher perceived neighborhood social cohesion was significantly associated with lower rates of depressive symptoms. Although the relationship was attenuated with the inclusion of behavioral and economic controls, it remained statistically significant. In additional analyses, we found that the mental health benefits of perceived neighborhood social cohesion do not vary by perceived neighborhood disorder.

**Conclusion:**

Our study emphasizes that strengthening neighborhood cohesion can enhance mental health as a structural‐level intervention for older adults with OSA. The mental health benefits of improving social cohesion in one's neighborhood of residence do not vary across levels of perceived physical disorder. By highlighting the psychosocial factors influencing health outcomes among older adults with OSA in sleep deserts, we provide insights into potential community‐wide interventions to improve sleep and mental health.

